# Edge computing-based proactive control method for industrial product manufacturing quality prediction

**DOI:** 10.1038/s41598-024-51974-z

**Published:** 2024-01-14

**Authors:** Mo Chen, Zhe Wei, Li Li, Kai Zhang

**Affiliations:** 1 School of Mechanical Engineering, Shenyang University of Technology, Shenyang, China; 2 Shenyang Innovative Design & Research Institute Co., Ltd., Shenyang, China

**Keywords:** Mechanical engineering, Engineering

## Abstract

With the emergence of intelligent manufacturing, new-generation information technologies such as big data and artificial intelligence are rapidly integrating with the manufacturing industry. One of the primary applications is to assist manufacturing plants in predicting product quality. Traditional predictive models primarily focus on establishing high-precision classification or regression models, with less emphasis on imbalanced data. This is a specific but common scenario in practical industrial environments concerning quality prediction. A SMOTE-XGboost quality prediction active control method based on joint optimization hyperparameters is proposed to address the problem of imbalanced data classification in product quality prediction. In addition, edge computing technology is introduced to address issues in industrial manufacturing, such as the large bandwidth load and resource limitations associated with traditional cloud computing models. Finally, the practicality and effectiveness of the proposed method are validated through a case study of the brake disc production line. Experimental results indicate that the proposed method outperforms other classification methods in brake disc quality prediction.

## Introduction

Since the beginning of the twenty-first century, industrial big data has experienced rapid development with the improvement of data collection and processing capabilities^1^. The centralized big data processing model centered around cloud computing can no longer support industrial data analysis. Its various drawbacks have become evident, such as difficulty integrating heterogeneous data from multiple sources, handling high broadband loads, and dealing with limited resources^2–4^. This is particularly critical for equipment management systems demanding highly real-time data processing. Suppose faults within the equipment are not detected at the earliest opportunity. In that case, it diminishes product processing quality and leads to even more significant losses across the entire industrial production line. In recent years, edge computing technology, based on industrial-grade intelligent hardware, has become a hot research field. Establishing a data bridge between production equipment and cloud-based systems achieves rapid sensing of equipment operating statuses in the industrial Internet of Things (IIoT) and enables intelligent adjustments. This advancement has propelled significant developments in intelligent systems and smart manufacturing^5^. The operating scope of edge computing technology includes downstream data from cloud services and upstream data from the Internet of Things services^6^. It is a novel computing model that performs computations at the network edge^7^.

Edge computing can be traced back to the content distribution network proposed by Akamai in 1998^8^. In 2013, the American scholar Ryan La Mothe first proposed “edge computing” in an internal report^7^. In May 2016, Professor Shi Weisong and his team from Wayne State University in the United States formally defined edge computing^9^. In the same year, China established the Edge Computing Consortium (ECC), which Huawei Technologies Co. Ltd. and the Shenyang Institute of Automation of the Chinese Academy of Sciences founded^7^. The consortium covers various fields, such as scientific research institutions and industrial manufacturing. In the case of the Industrial Internet of Things (IIoT), edge computing meets its requirements for real-time control and edge device security and privacy in practical applications, making it a direction for developing the IIoT industry. In their study, Shi Weisong et al.^6^ summarized the current situation and prospects of edge computing and provided a summary from the industrial Internet of Things perspective. Edge computing can address the real-time control of networked production and processing, edge device security and privacy, and localized processing of production data faced by the development of industrial IoT, and has advantages in improving performance, ensuring data security and privacy, and reducing operating costs in practical applications^6,7^.

**Figure 1:**
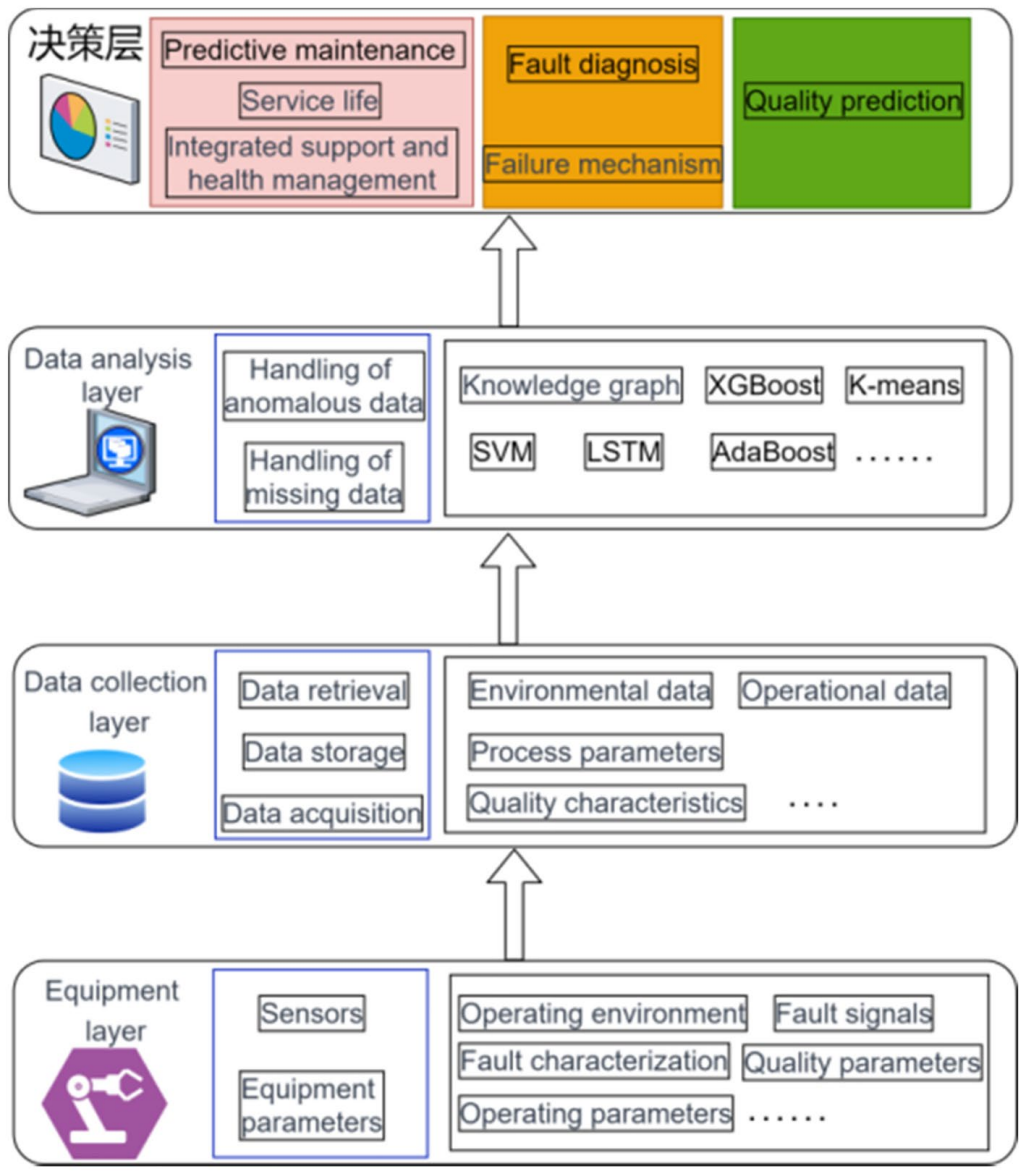
Research directions for equipment management.

Currently, research on equipment management mainly focuses on three directions: predictive maintenance^10–12^, fault diagnosis^13,14^, and quality prediction, as shown in Fig. 1**.** Equipment predictive maintenance refers to collecting operational data and environmental data during the operation of equipment, using big data and machine learning methods to predict the service life and damage of important components of the equipment, avoiding excessive maintenance of the equipment, reducing the failure rate of the equipment, and lowering the manufacturing cost of products. The main approach to equipment fault diagnosis is to establish the mechanism of equipment faults, study the relationships between various causes of faults, fault characterizations, and fault signals, in order to diagnose equipment faults quickly when they occur. Quality prediction is an essential means to reduce the probability of product quality problems and improve the qualification rate by analyzing the operating parameters of the equipment, obtaining quality characteristics based on equipment parameters, and monitoring and controlling the parameters of the equipment processing process^15^.

Product quality management is an essential issue in intelligent factory information services, and many scholars have elaborated on different aspects such as reliability^16^, helpful life^17^, and retrievability^18^. Among them, the prediction of product quality is also a hot topic. Product quality often requires specialized, expensive, and complex testing equipment, and the testing process can take a long time. Therefore, rapid, effective product quality prediction is significant for providing decision-making services to factory managers.

## Related work

The current research on quality prediction methods is mainly divided into two categories: model-based prediction methods and data-driven prediction methods. The main difference between the two methods lies in whether the design of the controller is based on the system model or only on the I/O data. In other words, whether the design of the controller involves the dynamic model of the system or not. If the system model is involved in the design of the controller, it is a model-based prediction method; otherwise, it is a data-driven prediction method^19^. From this perspective, it can be concluded that certain prediction methods, such as those reliant on neural networks, fuzzy control prediction techniques, and various other intelligent control prediction methods, are founded upon data-driven predictive approaches^20^. Many scholars have conducted extensive research and exploration on quality prediction. Table 1 summarizes the relevant papers.

**Table 1:** Research of related papers.

Type	Problem-oriented	Author	Main work
Model-based prediction methods	Accurate mathematical model is available; Mathematical model is inaccurate and involves uncertainties;	Wu et al.^21^	Develop tool wear assessment and life prediction models for real-time monitoring of drill bit wear
		Lin et al.^22^	Proposed a novel model-based approach for monitoring and predicting rotor-bearing system imbalance
		Marei et al.^23^	Introduced a transfer learning mechanism to design a hybrid CNN-LSTM model, enhancing the accuracy of predictions
		He et al.^24^	Calculating the cumulative variation in the assembly process using finite element method
Data-driven prediction methods	Mathematical model is inaccurate and involves uncertainties; Mathematical model is complicated with too high order or too much nonlinearity; Mathematical model is difficult to establish or unavailable;	Li et al.^25^	Implementing multi-source fusion of measured data and model data
		Liu et al.^26^	Develop a unified product quality prediction framework QTD based on end-to-end time series analysis
		Lee et al.^27^	Decision tree, random forest, support vector machine and other algorithms are used for quality prediction in casting processes
		Dong and Fen^28^	Using the XGBoost intelligent prediction model, the problem of precision prediction and control in the vehicle body assembly process has been solved
		Yu and Zhao^29^	An ordinal regression network model was applied to an actual industrial process

The current research on quality prediction methods is mainly divided into two categories: model-based prediction methods and data-driven prediction methods. The main difference between the two methods lies in whether the design of the controller is based on the system model or only on the I/O data. In other words, whether the design of the controller involves the dynamic model of the system or not. If the system model is involved in the design of the controller, it is a model-based prediction method; otherwise, it is a data-driven prediction method^19^. From this perspective, some prediction methods based on neural networks, fuzzy control prediction methods, and many other intelligent control prediction methods are based on data-driven prediction methods^20^. Many scholars have conducted extensive research and exploration on quality prediction. Table 1 summarizes the relevant papers.

From the existing research perspective, improving data acquisition and processing capabilities provides a foundation for data-driven quality control. It provides research ideas for the analysis of equipment operating data. This includes a model identification algorithm, proposing a multi-degree-of-freedom torsional vibration model for transmission systems, serving as a digital twin model for monitoring the remaining useful life of transmission system components^30^. Additionally, a method for predicting the quality of purifier carrier products is developed based on improved principal component analysis (PCA) and enhanced support vector machine (SVM). Other researchers have studied the mixed manifold learning and support vector machine algorithm based on optimized kernel functions (KML-SVM). They use support vector machines to classify and predict low-dimensional embedded data and optimize the kernel function of the support vector machine to maximize classification accuracy^31^. Using random forests for dimensionality reduction and analyzing key quality characteristics^32^. The principle of quality improvement in mechanical product development based on the Bayesian network can be used for the principle-empirical (P-E) model of quality improvement. It provides a method for learning the structure of the P-E model, and the quality characteristic (QC) relationship is determined by empirical data^32,33^. By analyzing the relationship between manufacturing resources and product quality status^34^, proposed a real-time quality control system (RTQCS) based on manufacturing process data, establishing the relationship between real-time product quality status and machining task processes^35^. A single-board computer and sensors were used to construct an edge device that can collect, process, store, and analyze data. Based on this, they developed a machine fault detection model using long short-term memory recurrent neural networks. Additionally, it is crucial to consider a real-time selection of the best model. In many cases, a simple probabilistic model can outperform more complex ones. Beruvides and colleagues achieved good drilling quality measurement and control results by employing the wavelet packet analysis method and fitting a statistical regression model^36^. Cruz and others proposed a two-step machine learning method for dynamic model selection, achieving favorable outcomes in predicting surface roughness during micro-machining processes and addressing complex cutting phenomena^37^.

These scholars have significantly contributed to quality prediction, but there are also some issues. Firstly, on a stable production line, the quantity of qualified products far exceeds the number of faulty products (imbalanced product quality labels). Therefore, the quality prediction problem becomes an imbalanced data classification issue. Secondly, the equipment environment during the production process is complex, with numerous equipment process parameters affecting the quality characteristics of the processed products. Selecting equipment process parameters helps reduce the dimensionality of prediction models. Thirdly, some cloud-based quality prediction methods may result in issues such as delay, high broadband load, and resource limitations. To overcome these shortcomings, this paper initially introduces edge computing into product quality prediction to ensure shorter response times and higher reliability. Then, a method for selecting quality-correlated parameters is designed. Finally, addressing imbalanced data classification problems is achieved by employing the Synthetic Minority Oversampling Technique (SMOTE) and Extreme Gradient Boosting (XGBoost). The scientific-technical contribution of this article:

1. Explored an edge computing-based framework for predicting the manufacturing quality of industrial products, offering guidance for flexible handling of industrial data.
2. The proposed is an active control method for quality prediction using SMOTE-XGBoost based on joint optimization of hyperparameters, applied in predicting manufacturing quality for industrial products to address the imbalanced data classification issue within product quality prediction. The experimental results validated the superiority of the proposed method.
3. Based on this paper’s proposed active control method for quality prediction, a selection and analysis of equipment process parameters for the brake disc production line was conducted using quality-correlated parameter selection, providing guidance and reference for the actual production and processing of brake disc products.

For modern manufacturing, ensuring reliable industrial product quality has always been crucial in enterprise manufacturing process control. Guided by data-driven proactive quality control, modern manufacturing enterprises can gather vast amounts of industrial product manufacturing process data and apply it across various models. However, these models must operate at sufficiently high processing speeds to meet the practical production needs. Hence, the introduction of edge computing technology plays a pivotal role. Deploying models to the edge of the production line according to the actual industrial environment and establishing an edge-side IoT platform allows for more effective processing and application of.

The remaining sections of this paper are organized as follows: section “Industrial product manufacturing quality prediction frame work” presents an edge computing-based framework for industrial product quality prediction. Section “Active control method for quality prediction” introduces a SMOTE-XGboost quality prediction active control method based on joint optimization hyperparameters. Following this, in section “Case study”, an experimental analysis of the processing quality of the brake disc production line is conducted based on the proposed quality prediction method, confirming the superiority of this approach and providing guidance and reference for actual brake disc production. Finally, section “Conclusion” provides a conclusion.

## Industrial product manufacturing quality prediction frame work

This section constructed an edge-computing architecture for the industrial Internet of Things and analyzed the application methods of existing architectures. This explains the necessity of deploying industrial product quality prediction models using edge computing methods and introduces the quality prediction method proposed in this study.

### Industrial internet of things for industrial production lines

**Figure 2:**
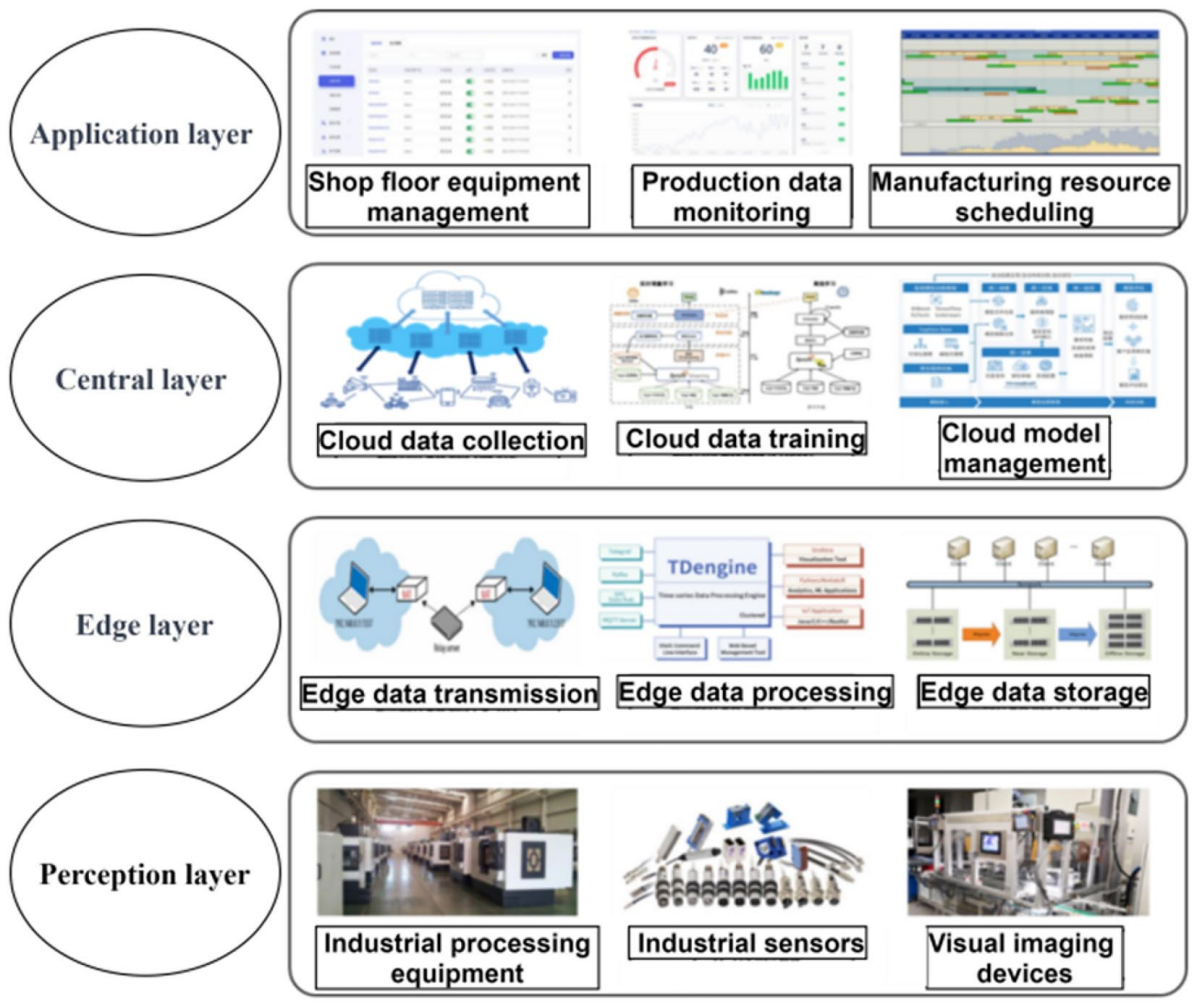
Industrial internet of things architecture for industrial production line.

To better manage the production line’s equipment operation status and product quality of the production line, and achieve real-timeproduct quality prediction, an industrial IoT architecture for the production line is established, as shown in Fig. 2. This is the basis for implementing industrial intelligence services.

This framework consists of four layers: perception layer, edge layer, central layer, and application layer.

### Quality prediction activecontrol method

**Figure 3:**
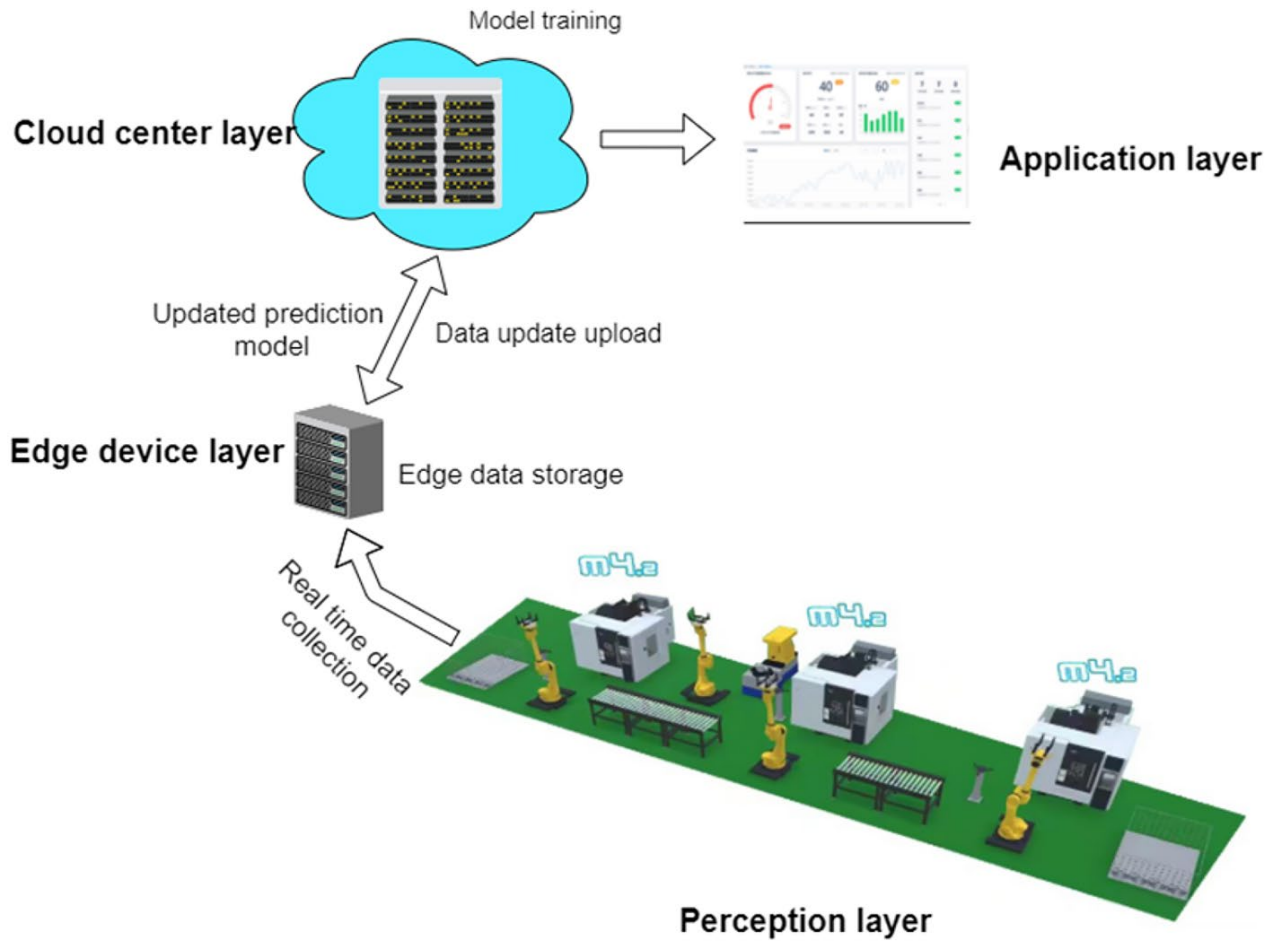
Quality prediction method supported by edge computing.

Figure 3 shows an example of industrial product manufacturing quality prediction based on edge computing. The data from the equipment side includes historical and real-time data and analyzes and describes its specific applications, while also analyzing the process parameter data during equipment operation.

#### Historical data

Historical data is mainly used for training the prediction model. The collected data is uploaded to the central layer through the perception layer for training the quality prediction model using machine learning algorithms. However, as the production process of products advances, the operating state changes over time. Therefore, the quality diagnosis and prediction model based on historical data is difficult to adapt to current production requirements. Some articles have also studied the update mechanism of predictive models^34,38^.

The complexity of manufacturing systems has led to the development of prediction methods that combine historical data and real-time measurement data, which are in line with the characteristics of edge computing technology.

#### Real-time data

Real-time data collected by the perception layer is transmitted to the edge layer, which undergoes preprocessing operations on the real-time data. Filtering the collected real-time data based on quality characteristic and then using the prediction model deployed on the edge device to make real-time judgments on product quality.

Simultaneously, the preprocessed data from edge devices is transmitted to the cloud center through the perception layer. As the data volume is reduced after preprocessing, it alleviates the bandwidth pressure and accelerates the transmission speed. For the received data, the central layer can update existing quality prediction models over time using incremental learning methods, addressing the issue of database updates in a time series.

#### Equipment process parameters

In existing research^15^, divided equipment process parameters into static process data, direct dynamic process data, and indirect dynamic process data based on their impact on the quality characteristics of the processed products to facilitate the application of equipment process parameters. Among them, static equipment process data refers to the type of equipment process data that generally does not change during the product processing process; direct dynamic process data refers to the equipment process data that changes dynamically during the product processing process, and the numerical changes directly reflect the product quality characteristics; Indirect dynamic process data refers to the equipment process data that changes dynamically during the processing process, but its changes do not directly reflect the product quality characteristics. Table 2 presents an example classification result of equipment process data^15^. Indirect dynamic equipment process data is the focus of this study.

**Table 2:** Classification results of equipment process data.

Type of equipment process data	Equipment process parameters
Static process data	Operator’s technical level, name, gender, age, etc. equipment type, power-on status, tool number, life, work piece count, etc. item code, item name, process name, etc
Direct dynamic process data	tool coordinate location, fault, etc
Indirect dynamic process data	spindle power, spindle current, spindle speed, feed rate, etc

### Introduction to the method

A proactive control method for quality prediction based on historical data is proposed, comprising two components: quality prediction and proactive control. The Active control methods refer to calculating the difference between the actual qualified rate of the produced product and the predicted qualified rate of products. If this difference exceeds a certain threshold, the edge computing layer will generate corresponding process adjustment control instructions and send them to the relevant processing equipment.

**Figure 4:**
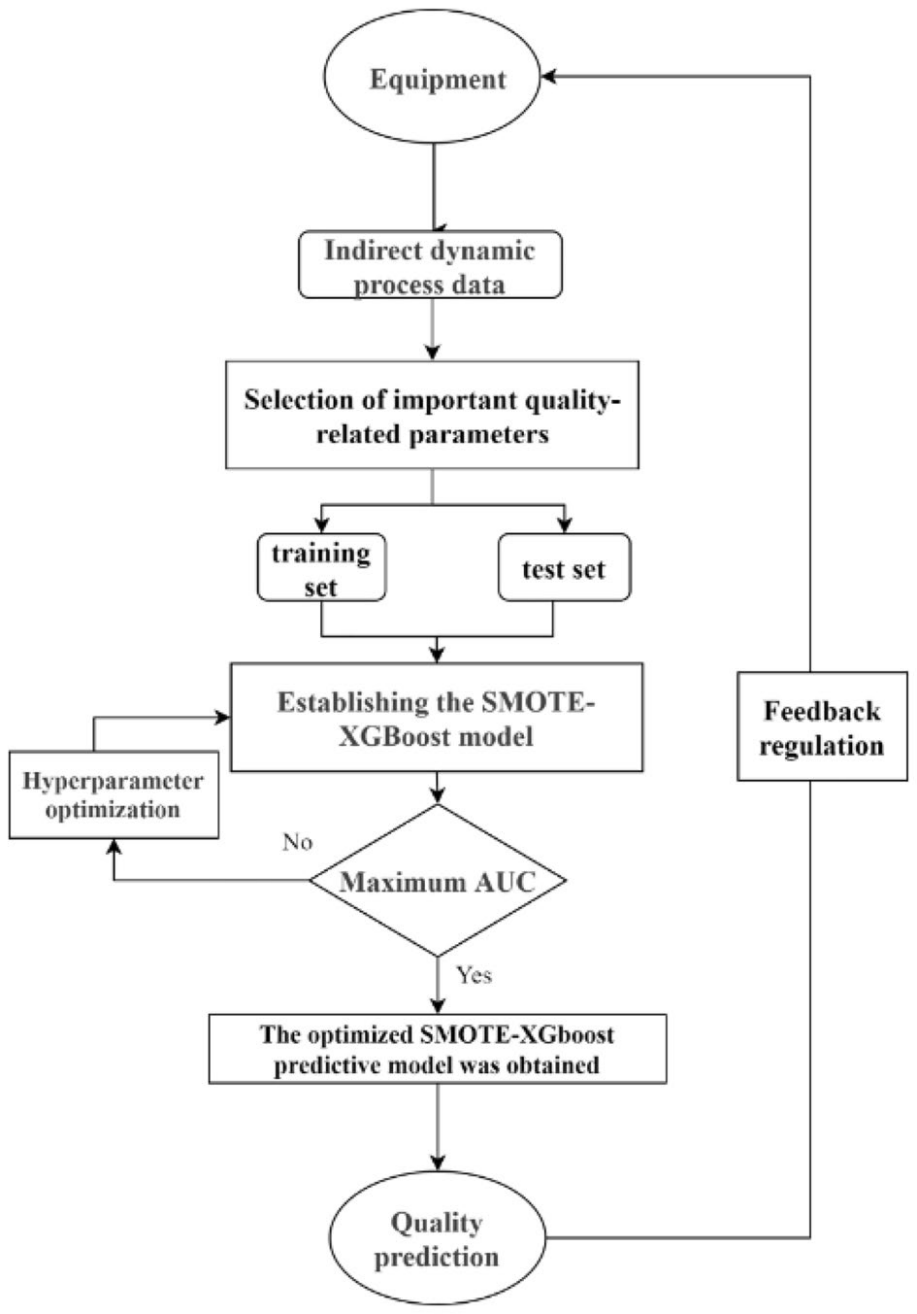
Shows the flowchart of the prediction method.

Figure 4 presents the workflow of this method. Firstly, indirect dynamic process data from production equipment is collected, and crucial quality-related parameters are computed using mutual information. These parameters are then selected based on their importance, followed by splitting the dataset into training and testing sets using stratified sampling. Subsequently, the SMOTE algorithm obtains a balanced dataset fed into the eXtreme Gradient Boosting (XGboost) for quality classification. Furthermore, a grid search method is applied for joint optimization of the hyperparameters of SMOTE and XGboost. Ultimately, the optimal quality prediction model is derived and utilized for product quality prediction. The details of this method are described in section “Active control method for quality prediction”.

## Active control method for quality prediction

This section first analyzed the product’s quality characteristics and selected criticalquality-related parameters with correlation coefficients greater than the set threshold based on the correlation coefficients of industrial product quality inspection results and quality-related parameters. Established the SMOTE-XGBoost quality prediction model and optimized the hyperparameters. Finally, the active control method for prediction.

### Analysis of quality characteristics

In product quality issues,this paper abstracts the product processing process as a manufacturing processing unit and the process of changing the product quality state as process characteristic data of processing quality. Additionally, it analyzesthe process parameter data during equipment operation.

**Figure 5:**
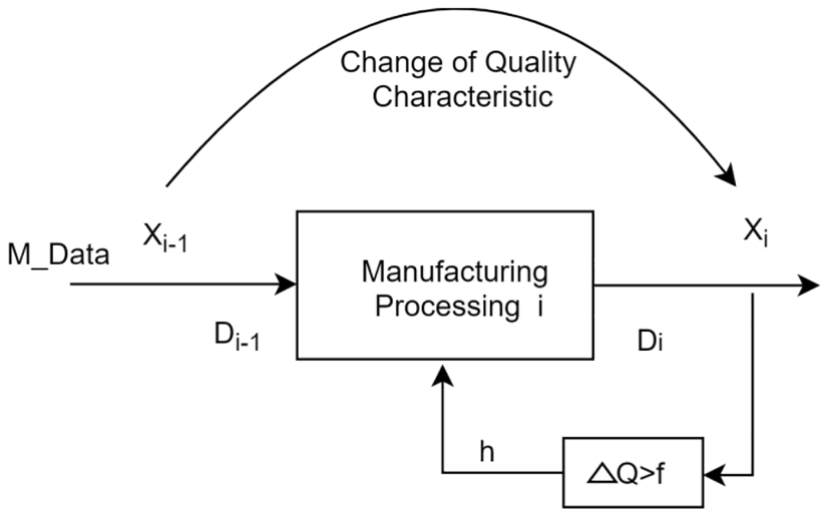
Manufacturing processing unit.

As shown in Fig. 5. In the manufacturing processing unit, ${X}_{i-1}$ represents the product state before the execution of the manufacturing processing unit;${X}_{i}$ represents the product state after the execution of the manufacturing processing unit; From the perspective of quality data, $M\_data$ refers to the resource processing data received by the manufacturing processing unit; ${D}_{i-1}$ represents the product quality state data before the manufacturing processing unit processes it; ${D}_{i-1}$ refers to the output product quality state data processed by the manufacturing processing unit; $\Delta Q$ represents the difference between the actual qualified rate of the calculated output product and the qualified rate of the industrial product containing the predicted results, and $f$ is the threshold. When the $\Delta Q$ value exceeds a certain threshold, the edge computing layer will generate corresponding process adjustment control instructions $h$ ,and send them to the relevant processing equipment, such as adjusting the spindle speed and feed rate^15^.

From the perspective of task execution, process $i$ refers to the process of transforming the quality characteristics of a product from state ${X}_{i-1}$ to state ${X}_{i}$ through a series of processing methods.

From the perspective of quality characteristics, the current quality characteristic ${X}_{i}$ is the result of the current process equipment processing the quality characteristic ${X}_{i-1}$ in the current environment^15^. The process of changing quality characteristics is the process of transforming input data into output data through its processing mechanism.

As the manufacturing processing continues, manufacturing quality-related parameter data is collected one by one at a fixed frequency. The type of equipment process data parameter set for collection is $i$, and each set of equipment quality-related parameter data collected is represented by an array, as shown in Eq. (1).

1 $$M\_Data=\left({m}_{1},{m}_{2}\cdots {m}_{i}\right)$$

In Eq. (1), $M\_data$ represents an array of equipment quality-related parameters collected at a certain moment. ${m}_{i}$ represents the $k$-th parameter of array $M\_data$. As time passes and the processing progresses, more and more data is collected, forming a matrix of quality-related parameter data as shown in Eq. (2).

2 $$\begin{array}{c}M\_Data=\left|\begin{array}{cccc}{m}_{11}& {m}_{21}& \cdots & {m}_{b1}\\ {m}_{12}& {m}_{22}& \cdots & {m}_{b2}\\ {m}_{13}& {m}_{23}& \cdots & {m}_{b3}\\ \vdots & \vdots & & \vdots \\ {m}_{1a}& {m}_{2a}& {m}_{1a}& {m}_{ba}\end{array}\right|\end{array}$$

### Selection of quality-related parameters

Throughout the production process of industrial goods, a large amount of data related to their quality is collected through the equipment perception layer, including quality inspection results and corresponding quality-related parameters. Including quality inspection results and corresponding quality-related parameters. Based on the quality inspection results and corresponding quality-related parameters, important rules for selecting quality-related parameters can be established, as described in section “Equipment process parameters”. This article selects the quality-related parameters that affect the indirectly dynamic equipment process data.

The selection rule of quality-related parameters mainly refers to selecting the key quality-related parameters with a correlation coefficient greater than a set threshold through the correlation analysis between the quality inspection results and the quality-related parameters in industrial product manufacturing. Formula for calculating the correlation coefficient $I\left({X}_{i}\right)$ between the quality inspection results and the quality-related parameters in industrial product manufacturing is:

3 $$I\left({X}_{i}\right)={\omega }_{0}{I}_{0}\left({X}_{i},{Y}_{0}\right)+{\omega }_{1}{I}_{1}({X}_{i},{Y}_{1})$$

4 $${I}_{0}\left({X}_{i},{Y}_{0}\right)=\sum_{x\in {X}_{i}}p\left(x,{Y}_{0}\right)log\frac{p(x,{Y}_{0})}{p\left(x\right)p({Y}_{0})}$$

5 $${I}_{1}\left({X}_{i},{Y}_{1}\right)=\sum_{x\in {X}_{i}}p\left(x,{Y}_{1}\right)log\frac{p(x,{Y}_{1})}{p\left(x\right)p({Y}_{1})}$$

where ${X}_{i}$ represents the $i$-th quality-related parameter, ${Y}_{0}$ represents the number of nonconforming industrial product manufacturing quality inspection results, and ${{\text{Y}}}_{1}$ represents the number of conforming industrial product manufacturing quality inspection results.$p(x,{Y}_{0})$ represents the joint distribution of ${X}_{i}$ and ${Y}_{0}.$ $p(x,{Y}_{1})$ represents the joint distribution of ${X}_{i}$ and ${Y}_{1}$. $p\left(x\right),p\left({Y}_{0}\right)$, and $p\left({Y}_{1}\right)$ are the probability distributions of variables ${X}_{i}$, ${Y}_{0}$, and ${Y}_{1}$,respectively. ${\omega }_{0}$ and ${\omega }_{1}$ are adjustment coefficients for data imbalance, with a sum of 1, generally determined based on the quality of the data samples obtained.

According to the correlation coefficient $I\left({X}_{i}\right)$ between the quality inspection results and the quality-related parameters in industrial product manufacturing, the importance of the features is sorted. Obtain a feature set $C$, where ${m}_{n}$ represents the $n$-th feature value.

6 $$C=\left\{{m}_{a}{m}_{b}\cdots \right.\left.{m}_{n}\right\}$$

### SMOTE-XGBoost algorithm for quality prediction

#### Data preprocessing based on SMOTE

According to the factory survey results, in the stable production line of brake discs, majority of the final quality is qualified, and only a small number of products have quality problems (unqualified products). The brake disc production line produces more than 1000 products per day, of which over 95% are qualified products. From a data mining perspective, this means that the input labels of the prediction model are imbalanced. Imbalanced label data is a common type of data that is widely present in various industrial fields.

**Figure 6:**
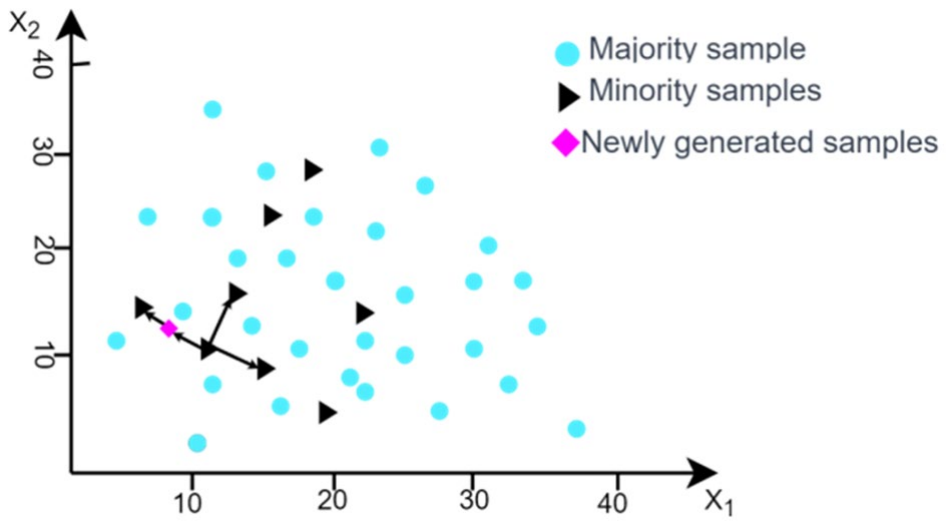
SMOTE oversampling algorithm principles schematic.

This article adopts the Synthetic Minority Oversampling Technique (SMOTE) algorithm to address the issue of imbalanced data. The core idea of the algorithm is to perform interpolation on the minority class samples in the dataset based on the k-nearest neighbor rule (as shown in Fig. 6 below). Generating more minority class samples as a result^39^. As the production dataset of brake discs is imbalanced, SMOTE is used in this chapter to balance the dataset. The main steps of the algorithm are as follows:

The dataset of brake disc processing collected by the production line is an imbalanced dataset. Based on the number of minority class samples ${N}_{min}$ and majority class samples ${N}_{max}$, the required number of synthesized samples $N$ is calculated:

7 $$N=\frac{{N}_{max}}{{N}_{min}}-1$$

For each unqualified product data sample (minority class) ${X}_{j}$, where ${X}_{j}{\in N}_{min}$. Select $k$ nearest neighbors ($k$ is usually set to 5) of the minority class sample ${X}_{j}$ randomly with the Euclidean distance as the measurement standard.

Assuming the selected neighboring point is ${X}_{K}$, the new synthetic sample point ${X}_{new}$ is generated according to the following formula.

8 $${X}_{new}={X}_{k}+rand\left(\mathrm{0,1}\right)\times \left({X}_{j}-{X}_{K}\right)$$

where $rand\left(\mathrm{0,1}\right)$ represents a random number between 0 and 1. Generate $N{*N}_{min}$ new minority class samples, merge them with the original data set to get a balanced data set. Then input them into XGboost for identification.

#### Predictive model based on XGboost

XGBoost is ensemble learning model framework based on gradient boosting algorithm, which was proposed by Dr. Tianqi Chen and his colleagues^40^. Compared with the traditional Gradient Based Decision Tree (GBDT), both are based on decision trees. However, XGboost effectively controls the complexity of the model and greatly reduces the variance of the model by using second-order Taylor expansion and adding regularization terms. The trained model is also simpler and more stable^41^.

Assuming that the input samples are $\left\{\left({x}_{1}{y}_{1}\right),\left({x}_{2}{y}_{2}\right),\cdots ,\right.\left.\left({x}_{n}{y}_{n}\right)\right\}$, The output of the XGboost model can be represented as the sum of $K$ weak learner outputs:

9 $${\widehat{y}}_{i}=\sum_{k=1}^{K}{f}_{k}\left({x}_{i}\right)$$

where ${f}_{k}\left({x}_{i}\right)$ represents the output of the $k$-th weak learner.

The model’s bias and variance determine the prediction accuracy of a model. The loss function represents the bias of the model, and to reduce the variance, a regularization term needs to be added to the objective function to prevent overfitting. The objective function comprises the model’s loss function $L$ and a regularization term $\Omega$ to suppress model complexity. The objective function to minimize in function space is:

10 $$L=\sum_{i}l\left({y}_{i}{\widehat{,y}}_{i}\right)+\sum_{k}\Omega \left({f}_{k}\right)$$

11 $$\Omega \left({f}_{k}\right)=\gamma T+\frac{1}{2}\lambda {\parallel \omega \parallel }^{2}$$

Here, $L$ represents the loss function, $\Omega \left({f}_{k}\right)$ represents the regularization function, $T$ is the number of leaf nodes, and $\omega$ is the weight value of leaf nodes. In the XGBoost model, most weak learns are based on Classification and Regression Trees (CART). Therefore, each round of optimization only focuses on the objective function of the $t$-th classification and regression tree based on the previous models.

12 $${\widehat{y}}_{i}^{\left(t\right)}={\widehat{y}}_{i}^{\left(t-1\right)}+{f}_{t}\left({x}_{i}\right)$$

13 $${L}^{\left(t\right)}=\sum_{i}^{n}l\left({y}_{i ,}{\widehat{y}}_{i}^{\left(t-1\right)}+{f}_{t}\left({x}_{i}\right)\right)+\Omega \left({f}_{t}\right)$$

Next, perform second-order Taylor expansion on the loss function of XGboos:

14 $${L}^{\left(t\right)}=\sum_{i}^{n}l\left[{{\text{g}}}_{i}{f}_{t}\left({x}_{i}\right)+\frac{1}{2}{h}_{i}{f}_{t}^{2}\left({x}_{i}\right)\right]\left({y}_{i ,}{\widehat{y}}_{i}^{\left(t-1\right)}+{f}_{t}\left({x}_{i}\right)\right)+\Omega \left({f}_{t}\right)$$

And in the above equation:

15 $${{\text{g}}}_{i}={\partial }_{{\widehat{y}}^{\left(t-1\right)}}l\left({y}_{i ,}{\widehat{y}}_{i}^{\left(t-1\right)}\right)$$

16 $${h}_{i}={\partial }_{{\widehat{y}}^{\left(t-1\right)}}^{2}l\left({y}_{i ,}{\widehat{y}}_{i}^{\left(t-1\right)}\right)$$

In which, ${{\text{g}}}_{i}$ and ${h}_{i}$ are the first-order and second-order derivatives of each sample on the loss function, respectively. Therefore, the optimization of the objective function can be transformed into the process of finding the minimum value of a quadratic function.

#### SMOTE-XGBoost with jointly optimized hyperparameters

(1) SMOTE-XGBoost model

The hyperparameter optimization methods mainly include grid search, random search, heuristic algorithms, and so on^42^. This article used the gridsearch method to optimize the above three hyperparameters, in order to obtain the optimal predictive model.

The Smote algorithm and the XGboost algorithm both have hyperparameters that need to be set before training the algorithm. The setting of hyperparameters affects the performance of predictive models. Previous research has mainly focused on the hyperparameters in classification or regression models. Therefore, consider Smote and XGboost as a whole and propose a joint optimization method for hyperparameters, called SMOTE-XGboost, to improve the performance of quality prediction models. Specifically, this paper focuses on the optimization of the hyperparameters $k$ in SMOTE (Number of nearest neighbors for selecting samples), $e$ in XGboost (Number of decision trees), and $T$ in XGboost (Number of leaf nodes). Selecting the maximum ${\text{AUC}}$ score as the optimization objective to obtain the best hyperparameters. The principle of joint hyperparameter optimization is as follows: Train the original SMOTE-XGboost model on historical data, which can be represented as:

17 $$M=SMOTE-XGboost(k,e,{\text{T}})$$

where $k$ represents the number of nearest neighbors selected in SMOTE. $e$ represents the number of decision trees in XGBoost, and $T$ represents the number of leaf nodes in XGBoost. Training process of the SMOTE-XGboost prediction model described in this article includes: To optimize the hyperparameters of the SMOTE-XGboost model with the goal of obtaining the maximum AUC score, the following formula is used:

18 $$ \begin{aligned} f = & \max \left[ {\mathop \sum \limits_{i = 1}^{t} L\left( {y_{i} ,\hat{y}_{i} } \right)} \right] \\ = & {\text{max}}\left\{ {\mathop \sum \limits_{i = 1}^{t} L\left[ {y_{i} ,SMOTE - XGboost(k,e,T|D_{1:t} )} \right]} \right\} \\ = & G(k,e,T|D_{1:t} ) \\ \end{aligned} $$

In the expression:${y}_{i}$ represents the true quality result; ${\widehat{y}}_{i}$ represents the predicted quality result; $\left[{y}_{i},SMOTE-XGboost(k,e,T|{D}_{1:t})\right]$ represents a quality prediction function; $G(k,e,T|{D}_{1:t})$ is a non-analytic function of the decision variable $k,e,T$. ${\text{L}}$ is the ${\text{AUC}}$ scoring formula; ${D}_{1:t}$ represents the first $t$ data points in the test set.

(2) Model evaluation indicators

To effectively evaluate the reliability of predictive models, comparative experiments of different algorithms are conducted using the coefficient of determination (${R}^{2}$) and the AUC as evaluation metrics to assess the relationship between predicted values and true values of the models. AUC is defined as the area enclosed by the coordinate axis under the ROC curve. It is a comprehensive performance classification indicator, which is commonly used to measure classification performance^31,43^. The higher the AUC, the better the algorithm performance.

Scoring formula for ${R}^{2}$:

18 $${R}^{2}=1-\frac{{\sum }_{i=1}^{N}\left({y}_{i},{\widehat{y}}_{i}\right)}{{\sum }_{i=1}^{N}{\left({y}_{i},\overline{y }\right)}^{2}}$$

In this expression, ${y}_{i}$ represents the true value, ${\widehat{y}}_{i}$ represents the predicted value, $\overline{y }$ represents the sample mean, and $N$ represents the sample size. A higher ${R}^{2}$ value indicates better performance. When the predictive model makes no errors, ${R}^{2}$ achieves the maximum value of 1.

Scoring formula for ${\text{AUC}}$:

20 $$AUC=\left[\sum_{i\in \,Positive\, sample\, se{\text{t}}}r(i)-\frac{M(M+1)}{2}\right]/M(N-M)$$

In this expression, $r(i)$ represents the ranking number of positive samples in the data set, $M$ represents the number of positive samples in the data set, and $N$ represents the total number of samples in the data set.

(3) Active control methods

In the actual production process, manufacturing process data of industrial products is first transmitted to edge computing nodes through Ethernet. The edge computing nodes use important quality-related parameter selection rules to filter and reduce data, and make real-time quality predictions for products as qualified or non-qualified based on the quality active prediction model deployed on the edge computing nodes.

Active control methods refer to calculating the difference between the actual qualified rate of the produced product and the predicted qualified rate of products. If this difference is greater than a certain threshold, the edge computing layer will generate corresponding process adjustment control instructions and send them to the relevant processing equipment, such as adjusting spindle speed, feed rate, etc.

Edge computing-based proactive control method for industrial product manufacturing quality prediction, It characteristics lie in the calculation formula for the difference $\Delta Q$ between the actual qualified rate of the produced product and the qualified rate of products with prediction results, which is as follows:

21 $$\Delta Q=\frac{{q}_{1}}{{Q}_{1}}-\frac{{q}_{2 }}{{Q}_{2}}$$

In the formula, ${q}_{1}$ and ${q}_{2}$ respectively represent the number of qualified products in the actual output, and the number of qualified products with prediction results; ${Q}_{1}$ and ${Q}_{2}$ respectively represent the total number of products in the actual output, and the total number of products with prediction results.

## Case study

This section takes the brake disc production line as an example to verify the practicality and effectiveness of the proposed method. The experiment consists of two parts: the selection of quality-related parameters and the classification results of the proposed method. Finally, the experimental results were analyzed.

### Experimental background

The data was obtained from a brake disc production line in a certain enterprise, which is mainly used to provide high-quality brake disc products for CRH (China Railway High-speed), urban rail transit, locomotives, and world-leading railway trains. In recent years, with the demand for low energy consumption and lightweight trains, the brake disc production line has undertaken the trial production tasks of new aluminum-based silicon carbide brake discs and carbon-ceramic composite brake discs, realizing the flexible switching between mass production and trial processing to adapt to R&D innovation and new market demands.

The brake disc is a component of the brake system that generates braking force to hinder the movement or motion trend of the vehicle. The surface of the brake disc requires high precision and must meet the qualified performance standards. The final quality inspection of the brake disc is tested by specialized magnetic particle inspection equipment and dynamic balancing equipment to determine whether it is qualified or not. This process takes a long time and the equipment is expensive. Therefore, using data-driven methods to predict the quality of brake disc products has the potential to replace specialized equipment, which can save equipment costs and inspection time.

The entire production line of brake disc machining includes production and processing equipment, inspection equipment, and each equipment is equipped with a data collection gateway, which collects data to the edge-side server for data storage and computing power. The historical data stored in the edge server is uploaded to the private cloud, and the proposed quality prediction model is trained in the private cloud center. The trained model is then deployed on the edge server, and real-time unmarked data is transmitted to the edge server via protocols such as OLE for Process Control Unified Architecture (OPC UA). The data is preprocessed on the edge server, such as removing abnormal values, and then the quality label is obtained through the quality prediction model on an industrial computer.

### Experiments and results

#### Qualitys correlation parameter selection

Some studies^44,45^ have pointed out that the equipment process data obtained from the processing equipment, including spindle power (P), spindle current (I), spindle speed (S), feed speed (F), and clamping force (N), were related to the changes of product quality characteristics in the processing process.

In order to validate the effectiveness of the proposed quality prediction method, historical data sets from the edge server were collected as the data source for overall quality prediction analysis. The data set includes 5 quality characteristics and 1 final quality label (qualified or fault product). The quality characteristics are all continuous random variables. Table 3 shows the specific quality characteristics of the partial samples. There are 1844 samples in the data set, including 1778 samples of qualified products and 66 samples of fault products. The imbalance ratio of the data set is about 26.9:1.

**Table 3:** The detailed description of quality characteristic.

Quality characteristics	Sample1	Sample2	Sample3	Sample4	Sample5	Sample6	Sample7
Including spindle power(P)/KW	7.34	7.25	7.15	7.35	7.28	7.17	7.15
Spindle speed(S) r/min	2041	2051	1807	2012	2037	2037	1794
Spindle current(I)/A	19.74	19.64	20.16	19.53	19.69	19.42	20.35
Feed speed(F) mm/min	1014	1014	1053	1007	1007	1026	1045
Clamping force(N) (kN)	21.43	21.43	21.37	21.39	21.48	22.53	22.13
Final quality	Qualified	Qualified	Fault	Qualified	Qualified	Qualified	Fault

**Figure 7:**
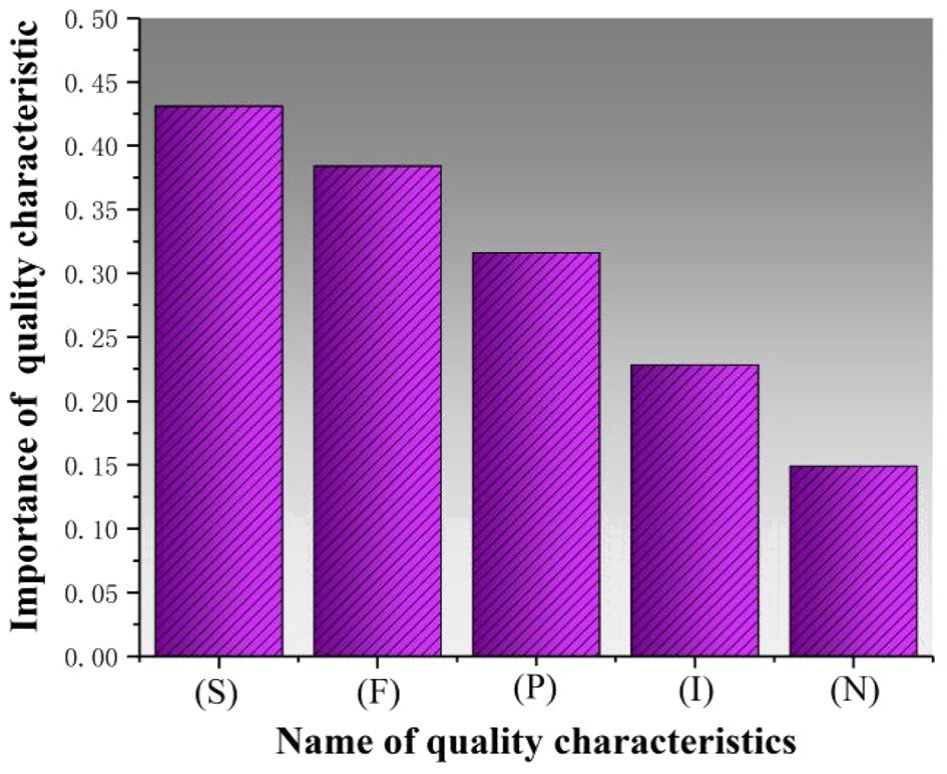
Importance of each quality characteristic.

As per the calculation method described in section “Selection of quality-related parameters”, calculated the importance of each quality feature and sorted them in descending order, as shown in Fig. 7, ultimately selected four quality features, including spindle speed (S) and feed speed (F), spindle power (P), and spindle current (I), and clamping force (N), to construct the prediction model.

#### Classification results with other machine learning methods

This paper conducted comparative experiments among different algorithms to validate the effectiveness of the proposed quality prediction model. The relationship between predicted and actual values was evaluated using coefficients (${R}^{2}$) and AUC as assessment metrics. All experiments in this study were deployed in a python3.6 environment and run on a desktop computer with an Intel Core i7 processor, 3.6 GHz, and 16 GB RAM.

**Figure 8:**
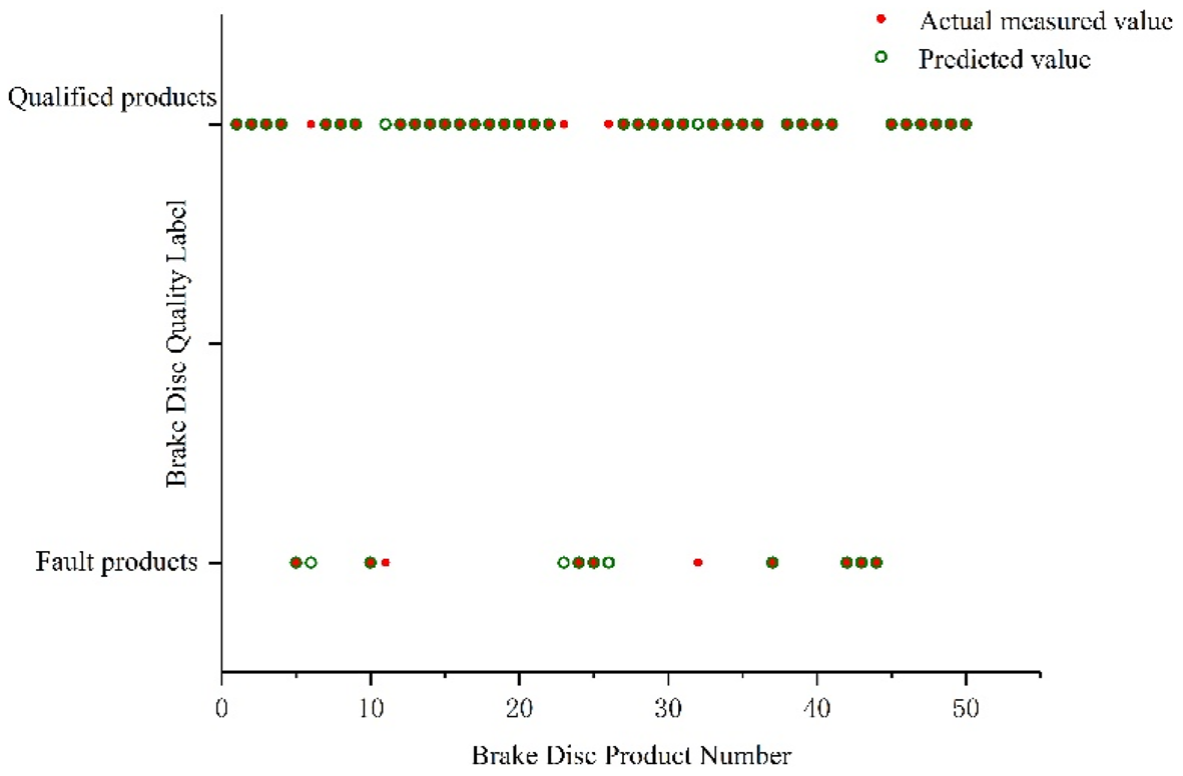
Predicted results of some test set data.

First, the data set was extracted based on the sorted quality features. The data description of the training set and test set is shown in Table 4. Apply the SMOTE oversampling strategy only in the training set to avoid over-optimism^38,46^. The data after SMOTE processing is shown in Table 5. Then, this text used the training set to build the SMOTE-XGBoost prediction model and used grid search to jointly optimize the hyperparameters of the brake disc quality prediction model (the hyperparameter optimization range is shown in Table 7). The final optimal values for each hyperparameter of the SMOTE-XGBoost were determined to be k = 6, e = 100, and T = 3. The optimized quality prediction model is named SMOTE-XGboost_t, and its prediction results on part of the test data set are shown in Fig. 8. This paper designed comparative experiments from the perspectives of classification algorithms and hyperparameter optimization to highlight the superiority of the proposed method.

**Table 4:** Description of each dataset.

Data set	Qualified product	Fault product	Total
Training set	1240	46	1286
Testing set	538	20	558
Total	1778	66	1844

**Table 5:** Description of each dataset after SMOTE.

Data set	Qualified product	Fault product	Total
Training set	1240	1240	2480
Testing set	538	20	558
Total	1778	1260	3038

(1) Comparison experiment of classification algorithms.

**Figure 9:**
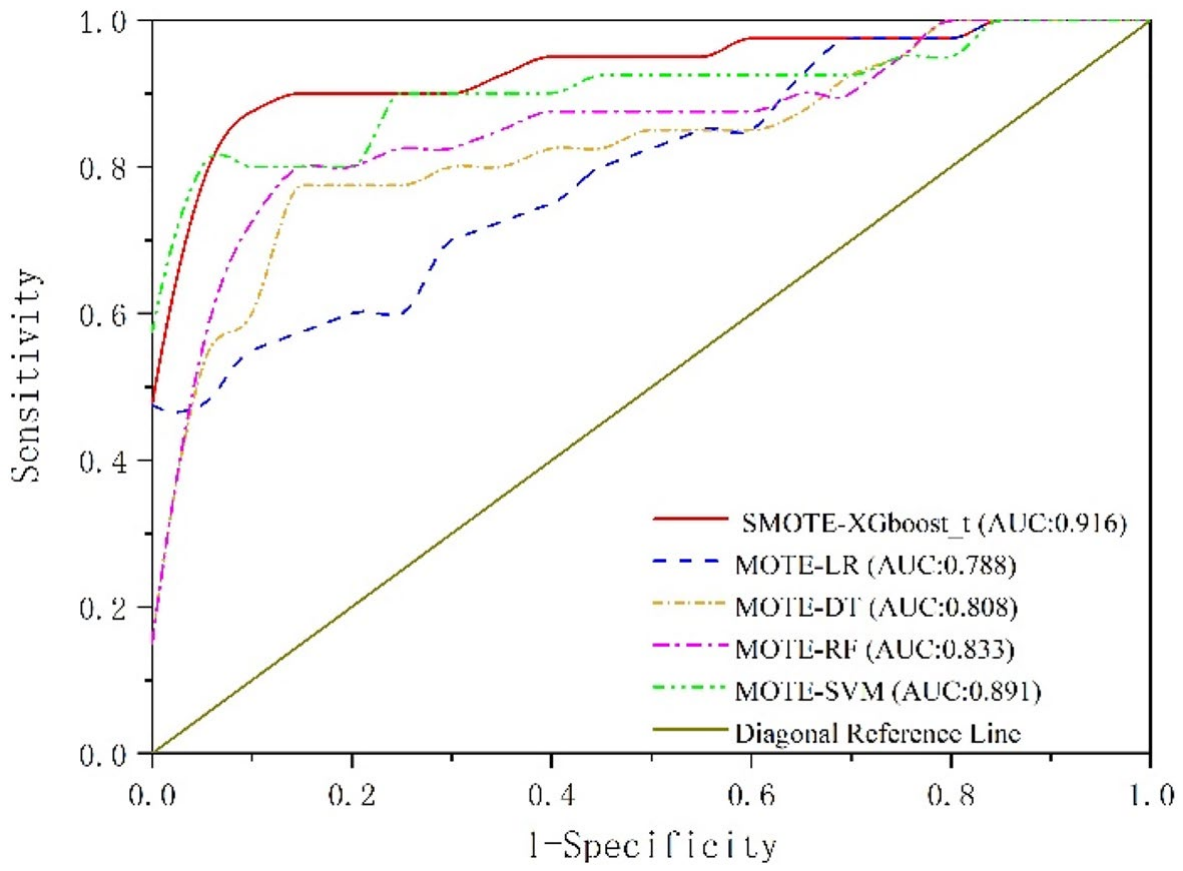
The ROC curve plot for the classification algorithms.

To verify the classification performance of the proposed method compared to other classification methods under the same criteria, this study used the same SMOTE method and compared the proposed method with other mainstream machine learning classification methods (Support Vector Machine, SVM; Logistic Regression, LR; Decision Tree, DT; Random Forest, RF). The experimental results are shown in Table 6, based on the table, as can be seen that the proposed SMOTE-XGboost_t method has slightly higher ${R}^{2}$ and AUC values compared to other classifiers in the experiment using the same SMOTE method. Moreover, the ROC curves of the model’s indicators are shown in Fig. 9. AUC is defined as the area enclosed by the coordinate axis under the ROC curve. From the figure, as can be seen that the AUC value of the proposed SMOTE-XGboost_t method is as high as 0.916, which indicates that the proposed method can effectively identify unqualified products and thus better predict the quality of brake discs.

**Table 6:** Comparison experiment of different classification methods.

Training model	*R*^2^	AUC
SMOTE-XGboost_t	0.897	0.916
SMOTE-LR	0.777	0.788
SMOTE-DT	0.885	0.808
SMOTE-RF	0.891	0.833
SMOTE-SVM	0.882	0.891

(2) Hyperparameter optimization comparative experiment

In addition, to investigate the impact of hyperparameter optimization on the model, this study conducted four different experiments:In the model named SMOTE-XGboost, the default values were used for the hyperparameters without any hyperparameter optimization; The hyperparameter $k$( Number of nearest neighbors for selecting samples) in SMOTE was optimized in the model SMOTE-XGboost_s; In the model SMOTE-XGboost_x, only the hyperparameters $e$ (Number of decision trees) and $T$ (Number of leaf nodes) in XGBoost were optimized; The last experiment involved joint optimization of the hyperparameters $k$ (Number of nearest neighbors for selecting samples), $e$ (Number of decision trees), and $T$ (number of leaf nodes) in both SMOTE and XGboost using grid search in the SMOTE-XGboost_t model. The optimal hyperparameters and optimization ranges for the predictive models in the four experiments are shown in Table 6, and the experimental comparison results are shown in Table 8.

Based on Table 7, see that in the SMOTE-XGboost_t model, the optimal value is 6 instead of the default value of = 5. This indicates that when integrating oversampling algorithms with traditional machine learning classification algorithms, there may be uncertainties in the prediction results due to the hyperparameters of the sampling model and the classification model. Therefore, optimizing the hyperparameters in both the SMOTE sampling algorithm and the XGboost classification model is beneficial to improve the quality prediction performance.

**Table 7:** The hyperparameters and optimization interval of models.

Model	k	e	T	Optimization interval
SMOTE-XGboost_t	6	100	3	k ∈ [3, 10] e ∈ [40,110] T ∈ [2,5]
SMOTE-XGboost_s	7	90	2	k ∈ [3,10], e = 90,T = 2
SMOTE-XGboost_x	5	80	4	k ∈ 5, e ∈ [40,110],T ∈ [2,5]
SMOTE-XGboost	5	90	2	k = 5, e = 90 T = 2

**Figure 10:**
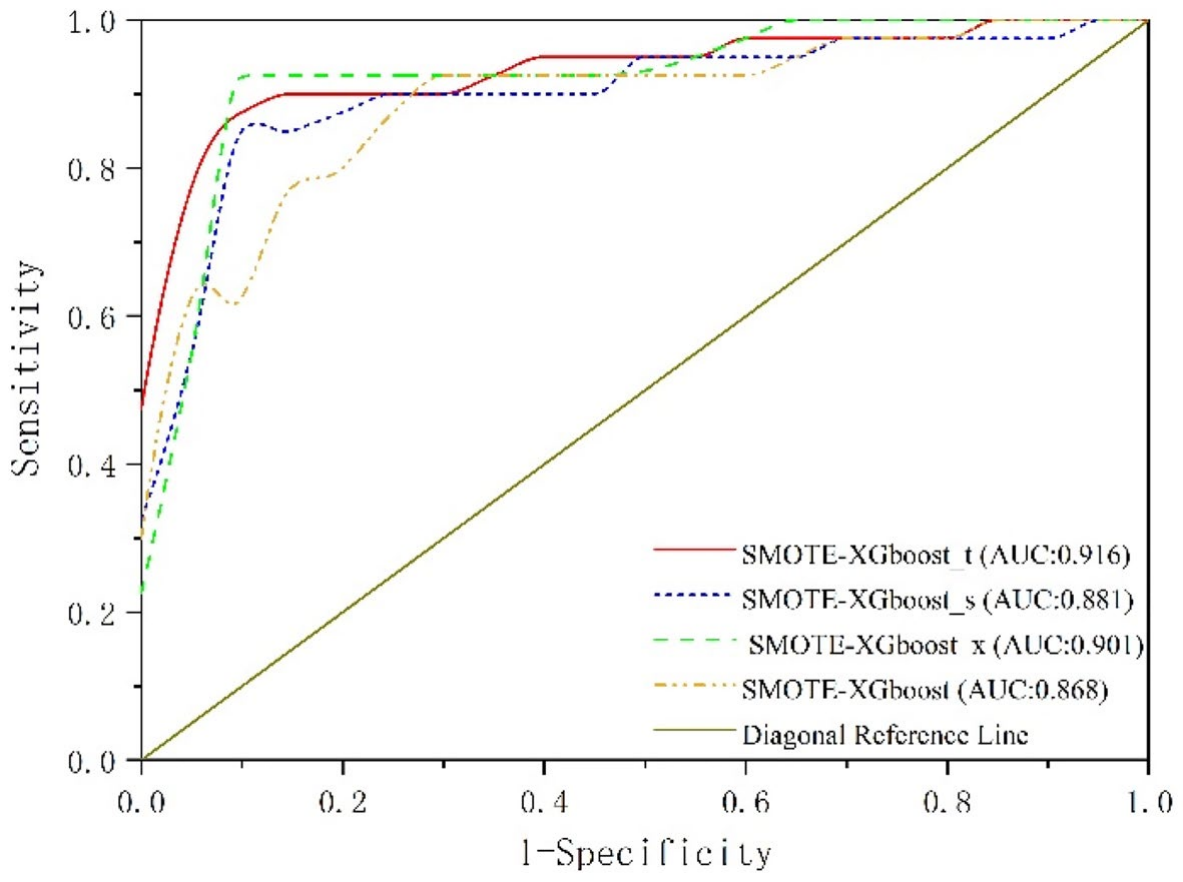
Comparison of ROC curves for hyperparameter optimization.

Analysis of the ROC curves for the four experiments based on AUC values is shown in Fig. 10. It can be observed that the SMOTE-XGboost_t and SMOTE-XGboost methods are slightly better than the other methods. SMOTE-XGboost_t had the best performance with an AUC value of 0.916.

The analysis from Table 8 shows that the proposed method performs better than other methods in terms of AUC and ${R}^{2}$ scores, indicating that the quality prediction model has a strong ability to identify the quality of brake discs after joint optimization of hyperparameters. Based on the actual operation of the factory, factory managers are more concerned about defective products than the large quantity of qualified products. Therefore, the method proposed in this paper has strong comprehensive prediction ability.

**Table 8:** Influence of hyperparameters optimization in models.

Model	R^2^	AUC
SMOTE-XGboost_t	0.897	0.916
SMOTE-XGboost_s	0.863	0.881
SMOTE-XGboost_x	0.881	0.901
SMOTE-XGboost	0.847	0.868

### Discussion

In terms of imbalanced data, Table 6 and Fig. 9 demonstrate that the SMOTE and XGBoost combination outperforms the combination of SMOTE with other classification algorithms Fig. 7 displays the importance of quality features; selecting these features is crucial for predicting and analyzing quality issues. Additionally, simultaneous investigation of hyperparameters in joint optimization included k (the number of nearest neighbors in SMOTE), e (the number of decision trees in XGBoost), and T (the number of leaf nodes in XGBoost). Table 8 and Fig. 10 indicate that the SMOTE-XGBoost method with jointly optimized hyperparameters can enhance classification performance.

This result also indicates that our proposed method contributes to addressing imbalanced data classification issues. ${R}^{2}$ and AUC are two widely used metrics in various classification problems. Additionally, AUC is a comprehensive metric that considers both qualified and defective products. Therefore, AUC is a more critical metric in unbalanced quality prediction scenarios and is widely used in various imbalanced classification problems.

Existing traditional industrial product manufacturing quality has long relied on passive analysis methods such as statistical monitoring. This method primarily involves testing the product quality using quality inspection equipment after the production and processing of the product. The limitations of this method lie in two aspects. Firstly, specific products require particular quality inspection equipment, which takes considerable time and involves expensive equipment. Secondly, it is impossible to forecast whether the product quality will be up to standard. When faults occur in equipment affecting product quality, there is no timely feedback for adjusting the equipment. So, rapid and efficient quality prediction methods can potentially replace specialized equipment, saving on equipment costs and testing time.

## Conclusion

This article proposes an Edge computing-based proactive control method for industrial product manufacturing quality prediction, addressing the issue of imbalanced data in the manufacturing process. Firstly, an edge computing-based framework for quality prediction in industrial product manufacturing was proposed. Secondly, a method for selecting quality-related parameters was designed, this provides insights into quality analysis problems. Finally, a SMOTE-XGboost quality forecasting active control method based on joint optimization hyperparameters is proposed to solve the problem of manufacturing quality forecasting of industrial products under category imbalance (Table 8).

This paper compared prediction algorithms based on five different classification methods under specific experimental conditions. The experimental results indicate that the proposed SMOTE-XGboost_t method slightly outperforms the other four classifiers in terms of ${R}^{2}$ and AUC metrics. This indicates that the proposed method has good performance in predicting the manufacturing quality of industrial products and detecting faulty products. Finally, the optimal values for each hyperparameter of SMOTE-XGboost were determined to be $k\hspace{0.17em}$= 6, $e\hspace{0.17em}$= 100, and $T\hspace{0.17em}$= 3, and the prediction results were better than those obtained through single hyperparameter optimization.

The research in this article enhances the capability for product quality control and provides intelligent information services for enterprises. However, there are still some issues that need further study. This paper only considered the product quality prediction results after processing in a single processing unit. Therefore, future research will focus on predicting product quality for multi-stage processing. Additionally, since the process-related data during manufacturing is incremental, another research direction involves addressing the issue of the source database of the quality prediction model in the edge computing scenario updating over time in the production line. This involves devising an incremental data training strategy for obtaining performance updates by training incremental data on the existing model.

## Data Availability

The datasets generated during and/or analysed during the current study are not publicly available due to [Information related to product processing] but are available from the corresponding author on reasonable request.
